# Sequential male mate choice under sperm competition risk

**DOI:** 10.1093/beheco/aru037

**Published:** 2014-03-18

**Authors:** Steven A. Ramm, Paula Stockley

**Affiliations:** Mammalian Behaviour and Evolution Group, Institute of Integrative Biology, University of Liverpool, Leahurst Campus, Chester High Road, Neston CH64 7TE, UK

**Keywords:** copulatory behavior, mate choice, mating effort, sex roles, sexual conflict, sexual selection, sperm allocation, sperm competition.

## Abstract

Male eagerness to mate is a central paradigm of sexual selection theory. However, limited sperm supplies mean that male sexual restraint might sometimes be favored under promiscuous mating. Here, we demonstrate dynamic plasticity in male mating effort when females are encountered sequentially under varying sperm competition risk. Rather than showing consistent eagerness to mate, male house mice (*Mus musculus domesticus*) instead tailor their mating effort according to likely reproductive payoffs. They are significantly less likely to mate when sperm competition is certain and potential reproductive payoffs low, but dramatically increase investment if they do choose to mate under such circumstances. By contrast, male mice are significantly more likely to mate in situations simulating extra-territorial copulations, where future risk of competition is high but so too are potential reproductive rewards. Differential mating propensity appears to be the primary mechanism by which male house mice allocate sperm adaptively under sperm competition risk because we find no evidence for facultative adjustment of sperm numbers per ejaculate or ejaculation frequency in response to female-related cues. We conclude that sequential male mate choice under sperm competition risk could be a widespread but often unappreciated mechanism of strategic sperm allocation.

## Lay Summary

New research shows that male house mice can be coy too. Male eagerness to mate is a central tenet of sexual selection theory, based on the expectation that male reproductive success is limited mainly by access to females. Here, we show that where sperm supplies are limited, males too can display considerable restraint in mating, targeting reproductive effort toward particular females.

## INTRODUCTION

Contrary to traditional views on sex roles (Darwin 1871; Bateman 1948), there is increasing evidence for the operation and evolutionary significance of male mate choice (Dewsbury 1982; Parker 1983; Wedell et al. 2002; Edward and Chapman 2011). Typically, such evidence comes from experiments in which males are presented with a simultaneous choice between 2 types of female and consistently exhibit a preference for mating with 1 type (e.g., Parker 1983; Schwagmeyer and Parker 1990; Simmons et al. 1994; Preston et al. 2005; Byrne and Rice 2006; Tudor and Morris 2009; Wong and McCarthy 2009; Edward and Chapman 2011; Tan et al. 2013). It is generally assumed that male mate choice is much less likely to occur in situations where potential mates are encountered sequentially (Barry and Kokko 2010; Edward and Chapman 2011), even though this will commonly be the case in nature. Recent evidence points to the strategic allocation of limited sperm reserves as one (cryptic) mechanism of male choice under such circumstances (e.g., Engqvist and Sauer 2001; Pizzari et al. 2003; Gillingham et al. 2009; Barbosa 2011; Lüpold et al. 2011; see also Spence et al. 2013). Such allocation may be facilitated by males adjusting the size or composition of their ejaculates (Parker 1998; Parker and Pizzari 2010) or via behavioral mechanisms such as varying ejaculation frequency (Preston and Stockley 2006) and sexual motivation (“The Coolidge Effect,” Dewsbury 1981a; e.g., Wilson et al. 1963; Dewsbury 1981b; Pizzari et al. 2003; Koene and Ter Maat 2007).

Theory predicting optimal sperm allocation decisions assumes that ejaculate investment is limited (Parker and Pizzari 2010). Applying similar logic to mating decisions, Dewsbury (1982) argued that an optimal strategy may not be to always inseminate as many females as possible. Rather, under conditions favoring male mate choice (Parker 1983; Schwagmeyer and Parker 1990; Edward and Chapman 2011), males might bias mating effort toward particular females and forego mating opportunities with others altogether, rather than allocating sperm among each available female. Although rarely considered, such male sexual restraint may be adaptive under competitive conditions where reproductive payoffs are low because limited sperm reserves can instead be targeted to more favorable mating opportunities (e.g., Schwagmeyer and Parker 1990). Thus, differential mating propensity can also be thought of as a form of strategic sperm allocation, but one where discrimination occurs as a precopulatory phenomenon (i.e., mate, don’t mate), rather than the differential allocation of sperm numbers per ejaculate or ejaculation frequency once the decision to mate has been made.

In this study, we examine both precopulatory and postcopulatory episodes of potential male selectivity in wild male house mice (*Mus musculus domesticus*) when females are encountered sequentially. Wild house mice live in social groups, typically consisting of several reproductive females resident within a territory that is defended by a single dominant male (Bronson 1979). However, male house mice are regularly exposed to a risk of sperm competition within their polygynous mating system because females often seek extra-territorial copulations that can result in multiply sired litters (Dean et al. 2006). In this study, we manipulate female-mediated cues of sperm competition risk (Parker 1998) and quantify the responses of male subjects with respect to mating propensity, copulatory behavior, sperm number per ejaculate, and total number of ejaculations. This experimental design allows us to test experimentally whether males are more or less willing to engage in copulations under varying conditions of sperm competition risk, as well as whether they adjust their ejaculates as predicted by current sperm competition theory (Parker 1998; Wedell et al. 2002; Parker and Pizzari 2010). Wild-derived male house mice were used as subjects to ensure natural socio-sexual responses to manipulated cues of sperm competition risk, and we utilized inbred laboratory mouse strains to provide stimulus females of closely similar within-strain phenotype (thereby minimizing variation in female traits other than those under experimental manipulation) and reliable sexual receptivity. Our results reveal dynamic plasticity in the mating effort of male house mice, providing novel experimental evidence of sequential male mate choice under sperm competition risk.

## MATERIALS AND METHODS

### Subjects

Wild male house mice were bred in captivity from a large outbred colony originating from populations in the northwest of England, UK, and were sexually mature (aged 7–8 months) at the beginning of the experiment. Female laboratory mice of 2 distinct laboratory strains (C57BL/6 and BALB/c) were obtained with previous breeding experience at ca. 6 months of age from Harlan UK Ltd (Bicester, UK). Male mice were always individually housed and female mice were housed in pairs (or sometimes individually when their cage mate was being used in an experimental trial), in standard rodent cages (48cm × 11.5cm × 12cm, M3; North Kent Plastic Cages Ltd, Kent, UK), with Corn Cob Absorb 10/14 substrate and paper wool bedding material, and ad libitum access to food (LabDiet 5002 Certified Rodent Diet) and water. Male house mice that are housed singly and exposed to female odors, as in this study, typically adopt aggressive territorial and scent-marking behavior similar to the behavior of dominant territorial males under natural conditions (Hurst 1990; Rich and Hurst 1998). Thus, by housing males singly and exposing them to female odors, our study was designed to stimulate normal behavioral responses of dominant male house mice occupying a defended territory with resident females. By exposing males regularly to female odors within their home cage, combined with direct exposure to these females, our aim was for subjects to become familiar with these females and respond when encountering them as if the females were normally resident in their territory. By contrast, when encountering females whose odors the subjects have not regularly encountered previously, we expect they should respond as if meeting a female from outside of their territory. All animals were maintained in the same animal room (with no other animals present) under the following controlled environmental conditions throughout the duration of the experiment: temperature 20–21 °C, relative humidity 45–65%, and a reversed 12:12h light:dark cycle (lights off at 08.00). Males were housed on a separate cage rack to females, with care taken to avoid potential odor contamination via transfer of soiled bedding between cages, backed up by a powerful room ventilation system (20 air changes/h).

### Experimental design

The experimental design is summarized in Figure S1 (in Supplementary Material). In a repeated measures design, each subject male was paired in a randomized order with females representing different cues of sperm competition risk based on mating status (previously unmated/previously mated), familiarity (familiar/unfamiliar), and stage of oestrus (early/late); full details of which are given below (see Manipulation of familiarity of females, Manipulation of female mating status, and Manipulation of female oestrus stage). In accordance with current sperm competition theory in relation to optimal sperm allocation (Parker 1998), we predicted that males would respond to social cues indicating that females are recently mated, unfamiliar, or at an early stage of oestrus because each of these conditions represents a relatively high risk of sperm competition. Different combinations of these cues produced 4 unique “treatments” of mating opportunities to males: 1) previously unmated, familiar strain, early oestrus females; 2) previously unmated, unfamiliar strain, early oestrus females; 3) previously unmated, familiar strain, late oestrus females; and (4) previously mated, familiar strain, late oestrus females. Subject males were allowed to mate on 2 separate occasions (with different females) in each of treatments 1–3 to permit separate measurements of sperm allocation (number of sperm transferred in first ejaculate) and ejaculation frequency (number of complete mating series culminating in ejaculation within a fixed 3-h time period), which cannot be measured simultaneously because in order to be counted, sperm must first be recovered from the female reproductive tract. By contrast, only ejaculation frequency was measured with 1 treatment 4 female (previously mated) per subject because in this case, it would not have been possible to distinguish between sperm from different males in the female reproductive tract and thus, we could not determine the number of sperm transferred by subject males.

#### Manipulation of familiarity of females

Consistent with the territorial social system of wild house mice (see Introduction), we used familiarity as a cue to manipulate perceived intra- or extra-territoriality of encountered females. To establish female familiarity, subject males were regularly exposed to odors from 1 of the 2 distinct female laboratory mouse strains (BALB/c or C57BL/6). Soiled bedding was transferred from a cage containing 1–2 laboratory female mice of one of these strains into the male’s home cage 3 times weekly, commencing 1 week before the start of the experiment and continuing for the duration of the experiment. In addition, 3 days prior to the start of the experiment, all subject males were allowed indirect contact (separated by cage bars) with 2 females of the same strain to which they had been familiarized for 90min. Half of the subject males were familiarized to BALB/c females and half to C57BL/6 females, to avoid any potential effect of the strain identity rather than familiarity per se. Because females of the same inbred laboratory strain should essentially smell the same, but the 2 strains employed come from 2 distinct genetic lineages and differ in their urinary odor profile (Cheetham et al. 2009), this approach allowed us to familiarize subject males simultaneously with a group of reliably sexually receptive stock females for use in mating treatments while minimizing variation due to female effects.

#### Manipulation of female mating status

Females that mated in an experimental pairing during the morning were subsequently paired again during the afternoon to test subject male responses based on female mating status. Toward the end of the experiment, we reentered subject males that had already completed copulations in all 7 mating treatments into the experiment, to maintain a supply of mated females. Because these trials provide additional information, we incorporated their outcome into the analysis of mating propensity, controlling for repeated measures on each male by using mixed models with male ID included as a random effect.

#### Manipulation of female oestrus stage

Oestrus duration in female house mice likely coincides with a single dark phase (Green 1966). We, thus, paired females either in the morning (early oestrus stage) or in the afternoon (late oestrus stage) of each experimental day to a female belonging to their familiar strain. These 2 groups thus also provide the control treatments required to test for effects of female familiarity and mating status, respectively, as described previously because the former can be compared with an unfamiliar, unmated female presented at the same time of day (morning) and the latter to a familiar, mated female also presented at the same time of day (afternoon, see Figure S1 in Supplementary Material).

### Mating trials

At the start of each experimental day, a pool of oestrus females was identified (based on vaginal cytology; Green 1966) for pairing with subject males based on a preallocated order of testing and ensuring at least a 7-day period between mating treatments for males to allow time for sperm replenishment (Stockley P, Edward DA, unpublished data; see also Jackson and Dewsbury 1979; Dewsbury 1983; Huck and Lisk 1985). All experimental pairings (up to 4 of which could be completed simultaneously) were conducted in high-sided enclosures (1.2 m × 1.2 m) supplied with food and water, and monitored remotely using CCTV equipment in an adjacent laboratory, with trials conducted in the morning (“early oestrus stage”) normally commencing between approximately 10.00 and 11.00 and trials conducted in the afternoon (“late oestrus stage”) between approximately 14.00 and 15.00 (the timing could not be fixed precisely owing to variation in the availability of experimental animals and the number of actual copulations on any one day). Mating pairs were monitored and separated either after the first ejaculation (to measure sperm allocation) or after a fixed period of 3h (to measure ejaculation frequency). This time period exceeds that normally taken for copulatory sequences involving 2 ejaculations in wild male house mice (median 74min and mean 95min; Estep et al. 1975) while permitting 2 full mating bouts to be accommodated within a single female oestrus period where necessary (to allow us to manipulate the time at which males were presented to females—see “Manipulation of female oestrus stage” for details). Male subjects were returned to their home cage, and female subjects were either removed from the experiment in order to measure sperm allocation (see Sperm allocation), returned to their home cage, or retained in the experimental apparatus to be used later on the same experimental day (i.e., in the previously mated female treatment group—see above). If the pair did not mate, subjects were removed after 3h and returned to their home cages. Males were given up to 5 consecutive opportunities to mate in each treatment. Males that failed to mate in their first 5 mating opportunities were removed from the experiment and not included in all subsequent analyses; all remaining males included in the study were responsive to females and showed normal sexual behavior. A small number of trials were terminated early due to signs of aggressive behavior between paired animals and recorded as a failure to mate.

In total, data were obtained from 174 experimental pairings yielding 85 copulatory bouts, each including at least 1 ejaculation, by 11 subject males. All males mated at least once in all 7 mating treatments, except for the previously mated female treatment group in which only 7 of the 11 males mated within the first 5 pairings with a previously mated female.

### Copulatory behavior

DVD recordings were used to quantify ejaculation frequency. Ejaculations are easily identified by a distinctive male “shudder” followed by a period during which the pair remains immobile. Prior to ejaculation, we also quantified instances of female resistance behavior to male approaches (i.e., female moving away, rearing up, or biting the male; see Table S2 in Supplementary Material) and 4 additional standard measures of male copulatory behavior in rodents: the number of mounts prior to the first intromission, intromission latency (time from the start of the trial to the first intromission), ejaculation latency (time from the first intromission to ejaculation), and the number of intromissions performed prior to ejaculation (Estep et al. 1975). Intromissions are the (usually multiple) bouts of penile insertion that occur prior to ejaculation, during each of which the male performs multiple intravaginal thrusts and between which the mating pair separates for a (usually brief) period (see Dewsbury 1972; Stockley and Preston 2004).

### Sperm allocation

Established procedures were used for measuring sperm allocation in the first ejaculate (Ramm and Stockley 2007, 2009a). Briefly, females were killed using an overdose of halothane and dissected after 10min to remove the female reproductive tract. This was macerated and placed in a Sterilin tube containing 2mL of 1% citrate solution for sperm to disperse for 10min. Sperm counts were then conducted on an Improved Neubauer hemocytometer using standard techniques (Ramm and Stockley 2007, 2009a).

### Statistical analyses

To analyze male mating propensity, we used generalized linear mixed models (GLMMs) with Laplace approximation and binomial error distribution (Bolker et al. 2009) with mating outcome (yes/no) as the response, male ID fitted as a random effect (to control for multiple observations per male), and female familiarity, mating history, and oestrus stage fitted as fixed effects. Treatment order for each subject was also fitted as a covariate to test whether mating propensity changed over the course of the experiment, but this effect was not significant (*P* = 0.2) and all main effects remained unchanged, so it is excluded from all models presented. Analyses were conducted using the lme4 package for R (version 2.15.2; R Development Core Team 2012) and JMP (version 10). Significance of fixed effects in GLMMs was assessed by comparison of models with and without the variable of interest included, using likelihood ratio tests, and minimal models determined by stepwise deletion.

Prior to analysis, data on sperm counts were log transformed to improve normality, and differences between treatments (familiar vs. unfamiliar, matched for mating status and oestrus stage; and early vs. late oestrus stage, matched for familiarity and mating status) were tested using paired Student’s *t*-tests. Male copulatory behavior measures were non-normally distributed and so the effect of female familiarity (familiar vs. unfamiliar), female oestrus stage (early vs. late), and female mating history (previously unmated vs. previously mated) were tested using Wilcoxon signed rank tests, again using paired data matched for the other 2 experimental treatment factors in each analysis.

### Ethics statement

The study was conducted according to UK legal and institutional animal research requirements. No Home Office Licence or local ethical review was required.

## RESULTS

### Males are less likely to mate with recently mated females

**Figure 1:**
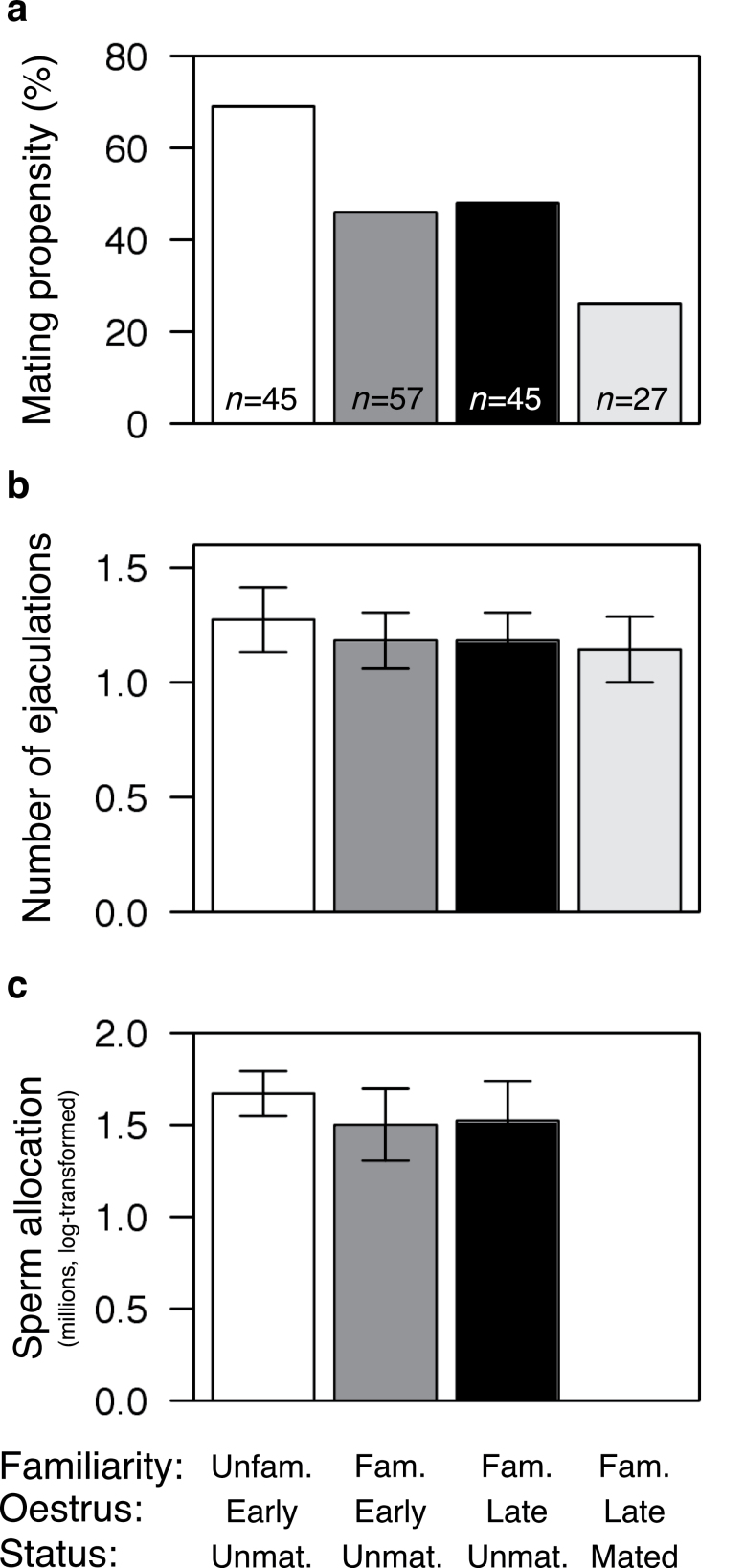
Male responses to 3 female-mediated cues of sperm competition (origin, mating history, and oestrus stage) at 3 different levels of reproductive effort: (a) mating propensity, (b) ejaculation frequency, and (c) sperm allocation per ejaculate. Males were significantly more likely to mate with unfamiliar females and significantly less likely to mate with previously mated females (a, see Table 1 for details) but did not significantly alter either their ejaculation frequency (b) or sperm allocation (c) according to these cues (see main text for test statistics). Bars in (a) represent the percentage of trials resulting in mating and in (b) and (c) means ± standard error of the mean.

**Figure 2:**
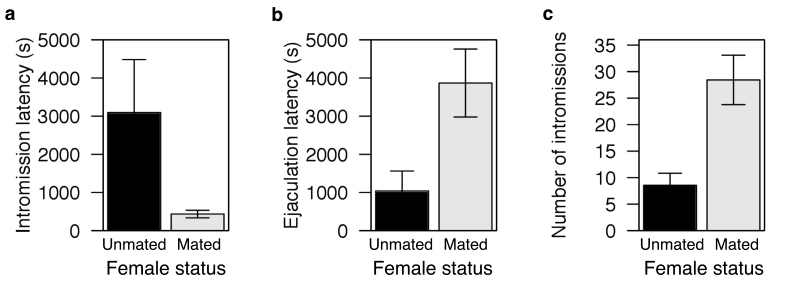
Effect of female mating history on copulatory behavior of male mice. Mating with a previously mated female induces a marked shift in copulatory behavior compared with matings with previously unmated females, involving (a) a significantly shorter intromission latency, (b) a significantly longer ejaculation latency, and (c) a significantly more intromissions prior to ejaculation. See main text for test statistics. Bars colored as per Figure 1.

Subject males were almost half as likely to mate with previously mated females compared with unmated females (26% of pairings with previously mated females resulted in mating, compared with 49% of pairings with previously unmated females of equivalent origin and oestrus stage; χ^2^ = 4.27, *P* < 0.05; Figure 1a, Table 1). The proportion of potential mating interactions involving evidence of female resistance did not differ significantly between treatment groups (Table S2 in Supplementary Material), indicating that differences in female sexual receptivity are unlikely to explain this reduced propensity to mate. Moreover, in cases where copulation did occur, it was initiated more rapidly with previously mated females (paired comparison of intromission latency for male subjects mating with mated and unmated females matched for familiarity and oestrus stage, Figure 2a: unmated females: 3094±1387; mated females: 435±99; Wilcoxon signed rank test, *S* = 14.0, degree of freedom [df] = 6, *P* = 0.016).

**Table 1:** GLMM to investigate factors affecting mating propensity of male mice, which is significantly influenced by both female familiarity and female mating status

Fixed effects	Estimate ± standard error	ΔAIC	χ^2^	*P*
(intercept)	−0.12±0.20			
Familiarity	0.92±0.38	4.08	5.97	0.014
Mating history	−0.93±0.48	2.26	4.27	0.045

AIC, Akaike information criterion. The analysis is based on data from 174 pairings (85 matings) involving 11 subject males, with male ID fitted as a random effect and a binomial error distribution. The final model revealed 2 predictors of male mating propensity: males are significantly more likely to mate with unfamiliar females (“familiarity” effect) but significantly less likely to mate with previously mated females (“female mating history” effect).

### Mating with previously mated females involves more effort

Like many rodents, house mice engage in multiple bouts of intravaginal thrusting—called intromissions—during copulation, between which males dismount and move away (Dewsbury 1972). Where females had previously mated and males initiated copulation, they then copulated for longer (paired comparison of ejaculation latency for male subjects mating with mated and unmated females matched for familiarity and oestrus stage, Figure 2b: unmated females: 1381±522; mated females: 2355±890; Wilcoxon signed rank test, *S* = −14.0, df = 6, *P* = 0.016) and performed substantially more intromissions prior to ejaculation (paired comparison of intromission number for male subjects mating with mated and unmated females matched for familiarity and oestrus stage, Figure 2c: unmated females: 8.57±2.26; mated females: 28.43±4.66; Wilcoxon signed rank test, *S* = −14.0, df = 6, *P* = 0.016). Again, this behavioral shift is not explained by an alteration in female behavior because there was no difference between treatments in the number of mounts required prior to the males first intromission (an indirect measure of female cooperation; median with unmated females: 2, median with mated females: 2, Wilcoxon signed rank test: *S* = −1.5, df = 6, *P* = 0.84), or in more direct measures of female cooperation (Table S2 in Supplementary Material).

### Mating is more likely with unfamiliar females

Here, we modeled encounters with intra- and extra-territorial females by pairing males with females from either a familiar or an unfamiliar female strain, respectively. Subject males in our study were significantly more likely to mate with unfamiliar females: 69% of pairings with unfamiliar (i.e., “extra-territorial”) females resulted in mating, compared with 46% with familiar (“intra-territorial”) females of equivalent oestrus stage and mating history (χ^2^ = 5.97, *P* < 0.02; Figure 1a, Table 1). This finding is not explained by female behavior because differences in familiarity were apparent only to males and were balanced between female strains (see “Experimental design” for details).

### Male mice do not adjust ejaculate size or frequency in response to female cues of sperm competition risk

Male mice did not alter ejaculation frequency (Figure 1b) or sperm allocation per ejaculate (Figure 1c) in response to female-mediated cues of sperm competition risk (Table 2), with the proviso that we could not measure sperm allocation in previously mated females (see “Mating with previously mated females involves more effort” for details). Similarly, when controlling for mating status and familiarity, the probability of mating by male mice did not differ according to female oestrus stage (*z* = 0.12, *P* = 0.9, Figure 1a, Table S1 in Supplementary Material), even though females in early oestrus presumably represent a relatively high risk of sperm competition compared with those in late oestrus because more time is available to the former to encounter further potential mates.

**Table 2:** Female-mediated cues of sperm competition risk do not affect ejaculation frequency (A–C), presented as the mean number of ejaculations per bout (and in brackets as the percentage of trials with double ejaculation) or sperm allocation per ejaculate (D and E)

	Male ejaculate allocation	Female cues of sperm competition risk		χ^2^ */t*	df	*P*
(A)	1. Mean ejaculation number per female (percentage of parings where male ejaculated twice)	Familiar	Unfamiliar	0.26	1	0.61
		1.18 (18%)	1.27 (27%)			
(B)		Early oestrus	Late oestrus	0	1	1.00
		1.18 (18%)	1.18 (18%)			
(C)		Unmated	Mated	0.05	1	0.83
		1.18 (18%)	1.14 (14%)			
(D)	2. Mean sperm number per ejaculate (×10^6^, ±standard error)	Familiar, early oestrus	Unfamiliar, early oestrus	0.75	10	0.47
		5.74±0.75	5.48±1.21			
(E)			Familiar, late oestrus	0.73	9	0.48
			5.45±0.94			

Statistical tests represent paired comparisons of subjects matched for other female cues of sperm competition risk; see main text for detailed description of these female-mediated cues. Test statistics for ejaculation frequency are based on chi-square tests of an association between treatment group and outcome (single or double ejaculation), and for sperm allocation on paired *t*-tests on the log-transformed sperm count data.

## DISCUSSION

Our study reveals a high degree of plasticity in male mating propensity under varying sperm competition risk, consistent with sequential male mate choice to optimize sperm allocation. Subjects were 1) significantly less likely to mate when sperm competition was certain and potential reproductive payoffs low, but 2) dramatically increased investment when they did mate under such circumstances, whereas 3) they were significantly more likely to mate with unfamiliar females, in situations simulating extra-territorial mating opportunities.

First, male house mice were significantly less likely to mate with recently mated females, even when no alternative potential mates were immediately available. How males recognize the mating status of potential partners was not investigated here, but it is likely that both odor-based cues of previous partners deposited on the female (Thomas 2010) and/or the physical presence of a copulatory plug (Ramm and Stockley 2007; Dean 2013) reveal mating status. Male mate choice under such conditions is consistent with low reproductive rewards of mating with previously mated females. Female mice that have recently mated represent a certain risk of sperm competition and offer a relatively low-potential reproductive reward for further prospective mates (compared with unmated females) due to a first male mating advantage in this species (Levine 1967). Male choice under such conditions may be partly related to the timing of the female’s first copulation, if fertilization rewards decline over time. Schwagmeyer and Parker (1990) reported that male 13-lined ground squirrels (*Spermophilus tridecemlineatus*) avoid copulating with previously mated females under natural conditions once a time threshold is exceeded whereby it should pay to instead search for alternative mating opportunities. However, given that mating is usually likely to entail a net benefit compared with not mating (the potential to sire some offspring versus none), our results also imply there must be limits to male mating capacity, which is central to the emergence of male mate choice (Dewsbury 1982; Edward and Chapman 2011).

It has recently been suggested that conditions favoring male mating selectivity may often arise in promiscuous lekking species (Saether et al. 2001; Bro-Jørgensen 2007), or more generally where competitively successful males achieve such high copulation rates that sperm depletion limits their mating capacity (Preston et al. 2001, 2005; Stockley and Bro-Jørgensen 2011). Our findings reveal that similar constraints can also apply when copulation is less frequent, as in the polygynous-promiscuous mating system of the house mouse (Bronson 1979), a species with relatively small testis size (Kenagy and Trombulak 1986) and limited sperm supplies (Huber et al. 1980). This suggests that male sexual restraint could be a common but relatively cryptic behavioral source of sexual conflict because females often benefit from mating multiply (Jennions and Petrie 2000), but males “can avoid mating without conspicuous resistance” (Bro-Jørgensen 2007).

Second, in cases where male mice did pursue copulation opportunities with previously mated females, a radical shift in copulatory behavior occurred, suggesting an “all-or-nothing” mating strategy. Under such conditions, male mice copulated for longer and performed substantially more pre-ejaculatory intromissions. Increased mating effort with apparently nonpreferred females appears paradoxical, but it may be necessary to maximize reproductive payoffs once the decision to pursue a mating opportunity has been made. Additional intromissions could have several potential benefits. For example, they might assist in dislodging copulatory plugs or other ejaculate components deposited by the first male (Hartung and Dewsbury 1978), provide additional stimulation to increase female fertility (de Catanzaro 1991) or male sperm allocation (Ramm and Stockley 2007), or conceivably inhibit female remating (Huck and Lisk 1986; cf., Løvlie et al. 2005). Regardless of function, this adjustment in male copulatory behavior reveals an increased time and energy cost of copulation with previously mated females. In combination with low potential fertilization rewards, this greater mating effort required presumably adds further disincentive for males to pursue mating opportunities with previously mated females, relative to unmated females.

Third, male mice in our study were significantly more likely to mate with unfamiliar females. Within the polygynous-promiscuous mating system of the house mouse, adult females typically not only reside and breed within the territory of a dominant male but can also move between male territories in pursuit of multiple mating opportunities (Bronson 1979; Singleton and Hay 1983). Unfamiliar females encountered by males will, therefore, typically come from outside of the male’s territory and thus present an elevated risk of sperm competition. This is because it is likely that such females will mate with the dominant male from the territory where they normally reside, but it also means that, from the point of view of the male encountering an unfamiliar female, such encounters could offer relatively “low-cost” mating opportunities, with for example, no subsequent parental investment (Dewsbury 1985). Because extra-territorial or unfamiliar females are likely to visit a male’s territory unpredictably, perhaps the most likely explanation for our results is that it always pays to pursue mating opportunities with such females more vigorously in order to maximize the chances of achieving mating success within a potentially limited timeframe.

An increased propensity to mate with unfamiliar females could also indicate that the benefits of extra-territorial copulations outweigh costs of elevated sperm competition risk. When mating with unfamiliar females in our experimental setup, males could potentially benefit from a first male mating advantage (they encountered as-yet unmated extra-territorial females who might normally be expected to go on to mate with the dominant male in their home territory) and more generally from additional postcopulatory mechanisms by which sperm use or viable offspring numbers may be biased in their favor (Eberhard 1996; Zeh and Zeh 2003). Where females from outside a male’s territory are relatively genetically dissimilar to him, successful copulations with unfamiliar females could also offer additional reproductive gains due to heterosis or increased offspring heterozygosity (Thoss et al. 2011). Hence, depending on the relative cost to reproductive success of sperm competition, males may often favor extra-territorial, unfamiliar, or novel females as mates (e.g., Kelley et al. 1999; Tokarz 2008; Tan et al. 2013; see also Spence et al. 2013). Finally, we note that our results concerning females differing in familiarity encountered sequentially over relatively long timescales are distinct from the well-established Coolidge effect described in many rodents (e.g., Wilson et al. 1963; Dewsbury 1981b) and some other animals (e.g., Pizzari et al. 2003; Koene and Ter Maat 2007), which is the restoration of sexual interest by a sexually satiated male when presented with a novel female (Dewsbury 1981a; see also Pierce et al. 1992). Nevertheless, both the Coolidge effect and the behavioral plasticity we have described here likely stem from a common cause, namely the benefit to males of conserving sperm supplies to invest in the most propitious mating opportunities (Dewsbury 1982).

Despite marked differences in mating propensity and copulatory behavior, male mice did not alter ejaculation frequency or sperm allocation per ejaculate in response to female-mediated cues of sperm competition risk. Strategic allocation of sperm or other ejaculate components in response to female-mediated cues, termed cryptic male choice, has been demonstrated in some previous studies, for example, in insects (e.g., Wedell 1992; Engqvist and Sauer 2001; Barbosa 2011; Lüpold et al. 2011) and birds (e.g., Pizzari et al. 2003, 2004; Gillingham et al. 2009). However, our negative findings here (cf., Pizzari et al. 2004) are consistent with results of previous tests in male house mice that found no evidence for adaptive variation in the number of sperm ejaculated in response to cues of immediate sperm competition risk mediated by the presence or absence of cues of a rival male (Ramm and Stockley 2007, 2009a). Rather, a previous study demonstrated that male mice plastically adjust overall investment in sperm production according to population-level cues of sperm competition risk (Ramm and Stockley 2009b), implying that they may be tailoring their ejaculates to average rather than immediate competitive conditions.

## CONCLUSIONS

We have identified sequential male mate choice as a mechanism of strategic sperm allocation in house mice. Specifically, we demonstrate the differential propensity of males to mate with females presenting various cues of sperm competition risk. The unexpected degree of plasticity in male mating effort revealed by our experiment challenges traditional sex roles (Darwin 1871; Bateman 1948), where a “great eagerness of the male” is generally expected in the context of sexual selection and mating (Darwin 1871, p. 240), and often leads to sexual conflict over optimal mating rates (Holland and Rice 1998; Arnqvist and Rowe 2005; Parker 2006). Male house mice instead demonstrate striking and predictable variation both in their propensity to mate with different females and in their copulatory behavior when doing so. By tailoring their mating effort to the potential risks and rewards of mating, the overall effect is to exhibit considerable sexual restraint. Given that the circumstances modeled by our experiments occur commonly in natural populations of diverse animal taxa, our results suggest that evidence of sequential male mate choice may often be overlooked in experimental and field studies of sexual selection, and that this likely represents an important but often unappreciated component of strategic sperm allocation.

## SUPPLEMENTARY MATERIAL

Supplementary material can be found at http://www.beheco.oxfordjournals.org/

## FUNDING

This work was supported by grants from the Leverhulme Trust (Grant F/00025/W) and the Natural Environment Research Council (E/I013008/1), United Kingdom. 

## Supplementary Material

Supplementary Data
