# Supplementary material for: Sequential male mate choice under sperm competition risk
Source: Behav Ecol. 2014 Mar 18;25(3):660–7. doi: 10.1093/beheco/aru037 (PMC4014308; doi:10.1093/beheco/aru037)
Supplement: Supplementary Data [file supp_25_3_660__index.html]

Sequential male mate choice under sperm competition risk — Sequential male mate choice under sperm competition risk — Supplementary Data 

# Sequential male mate choice under sperm competition risk

## Supplementary Data

Data files

**Files in this Data Supplement:**

- Supplementary Data - Supplementary Data
