# Supplementary material for: Sequential male mate choice under sperm competition risk
Source: Behav Ecol. 2014 Mar 18;25(3):660–7. doi: 10.1093/beheco/aru037 (PMC4014308; doi:10.1093/beheco/aru037)

# Supplementary Material

# Table S1

Generalized linear mixed model to investigate factors affecting mating propensity of male mice. Compared to Table 1, the full model below includes two additional, non-significant effects of “female oestrus stage” (i.e. there was no evidence that time of day affected the outcome of the mating trial) and “mating order” (i.e. suggesting male mating propensity did not change consistently over the course of the experiment).

Fixed effects Estimate ± SE z *P*

(intercept) -0.48 ± 0.36 -1.32 0.23

female origin 0.91 ± 0.42 2.14 <0.05

female mating history -1.07 ± 0.54 -1.99 <0.05

female oestrus stage 0.05 ± 0.41 0.12 0.90

mating order 0.04 ± 0.03 1.32 0.19

Table S2

In order to test whether differential resistance by females could account for the differences in mating propensity observed in trials involving either unmated or mated females, we performed detailed behavioural analyses of DVD recordings from 31 experimental trials where the pair did not mate, representing the first time each of 21 females was paired in one or both of the two treatment groups differing in mating status but matched for familiarity and oestrus stage. We classified interactions between the male and the female during the first 30 minutes of each trial, recording the number of times the male sniffed at the female without overt female resistance; the number of times the male mounted the female without overt resistance; and the number of times a male approach of the female resulted in behaviour that could indicate female resistance (the female rearing up on her hind legs, turning away from the male or attempting to bite the male). The latter two categories represent potential mating attempts by the male, and seven trials were excluded from further analysis due to there being no mounts (without resistance) or approaches with resistance within the first 30 mins, making female resistance behaviour impossible to estimate. To test for differences across treatments, we performed general linear mixed models, fitting female ID as a random effect to account for repeated measures on some females and treatment group (mated, unmated) as a fixed effect. These analyses revealed that there were no differences between the mated and unmated treatment groups in bouts of male sniffing without overt resistance (a) or in the number of male mounts without resistance (b). There was a non-significant trend towards more approaches with resistance in the mated treatment group (c). However, the proportion of potential mating interactions involving evidence of female resistance did not differ significantly between treatment groups (d). Analyses were performed in JMP (v. 10).

Dependent variable *t P*

a) # sniffs, no resistance -1.79 0.17

b) # mounts, no resistance 0.95 0.35

c) # approaches with resistance 2.06 0.08

d) prop (c / (b+c)) 0.19 0.85

**Fig. S1.** Summary of the experimental design, as described in the main text (see “Experimental Design” in Materials and Methods).


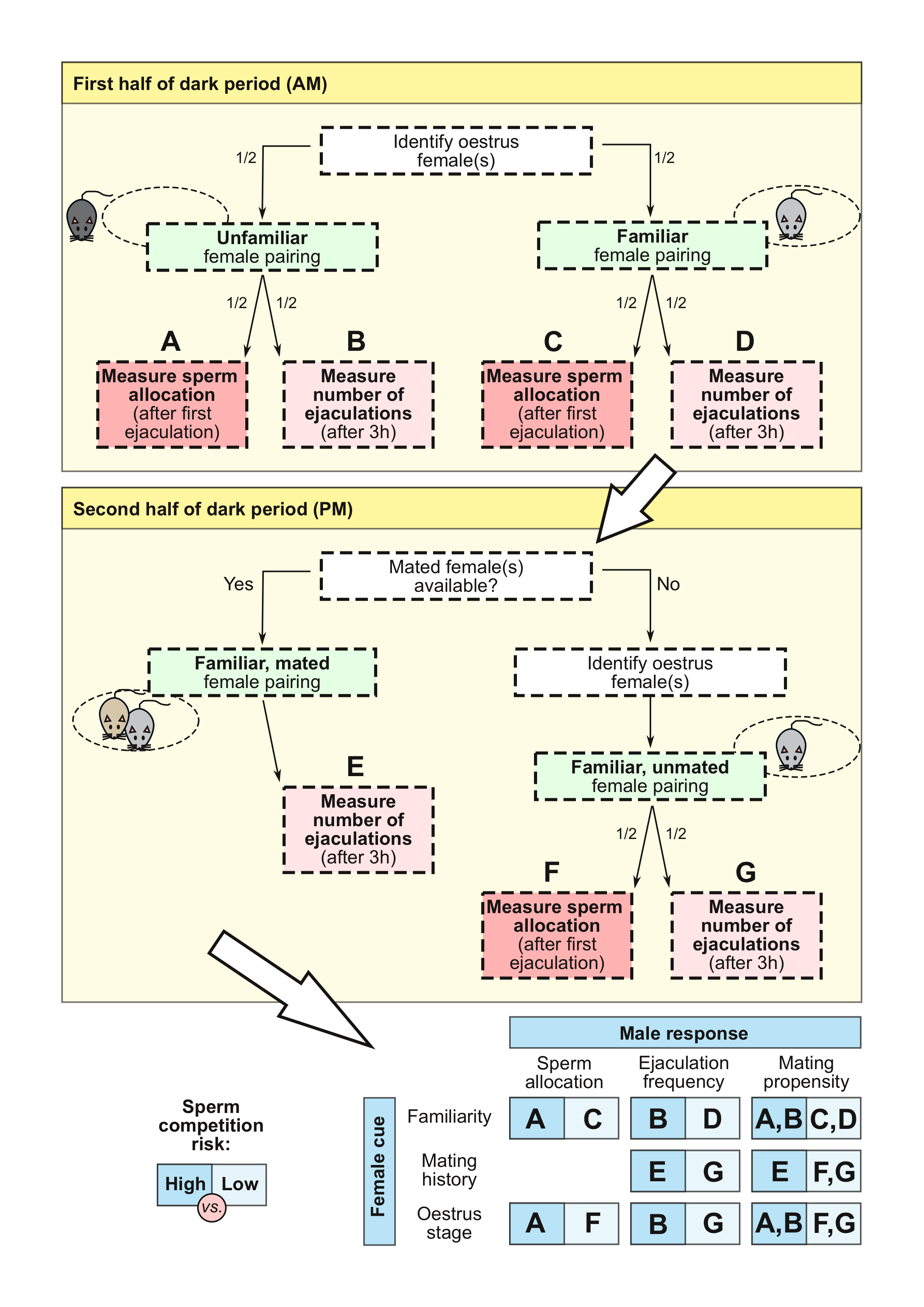

Supplement: Supplementary Data [file supp_aru037_RevisedSuppMaterial.doc]
